# Continuous Flow-Constructed Wetlands for the Treatment of Swine Waste Water

**DOI:** 10.3390/ijerph15071369

**Published:** 2018-06-29

**Authors:** Abasiofiok M. Ibekwe, Shelton E. Murinda

**Affiliations:** 1 USDA-ARS, U.S. Salinity Laboratory, 450 W. Big Springs Rd, Riverside, CA 92507, USA; 2 Department of Animal and Veterinary Sciences, California State Polytechnic University, Pomona, CA 91768, USA; semurinda@cpp.edu

**Keywords:** constructed wetland, contaminants, *E. coli*, REP-PCR, pollution

## Abstract

The microbiological quality of treated waste water is always a concern when waste water is disposed to the environment. However, when treated appropriately, such water can serve many purposes to the general population. Therefore, the treatment and removal of contaminants from swine waste water by continuous flow-constructed wetlands involves complex biological, physical, and chemical processes that may produce better quality water with reduced levels of contaminants. Swine waste contains *E. coli* populations and other bacterial contaminants originating from swine houses through constructed wetlands, but little is known about *E. coli* population in swine waste water. To assess the impacts of seasonal variations and the effect of the wetland layout/operations on water quality, *E. coli* isolates were compared for genetic diversity using repetitive extragenic palindromic polymerase chain reaction (REP-PCR). None of the isolates was confirmed as Shiga toxin producing *E. coli* O157:H7 (STEC); however, other pathotypes, such as enterotoxigenic *E. coli* (ETEC) were identified. Using a 90% similarity index from REP-PCR, 69 genotypes out of 421 *E. coli* isolates were found. Our data showed that the *E. coli* population was significantly (*p* = 0.036) higher in November than in March and August in most of the wetland cells. Furthermore, there was a significant (*p* = 0.001) reduction in *E. coli* populations from wetland influent to the final effluent. Therefore, the use of continuous flow-constructed wetlands may be a good treatment approach for reducing contaminants from different waste water sources.

## 1. Introduction

Continuous flow-constructed wetland is a natural process for the treatment of waste water [1,2,3]. It is an alternative to conventional technologies for wastewater treatment [2]. In places with concentrated animal feeding operations (CAFOs), such as North Carolina [4], current management practices for swine waste involve long-term storage in ponds where it is left to evaporate, percolate into groundwater, or sprayed onto crops and/or disposal lands. Swine waste is high in dissolved and particulate organic matter, ammonia and organically bound phosphorus and nitrogen as well as biological oxygen demand, and other contaminants. Swine waste may also contain *E. coli*, protozoan parasites such as *Cyrptosporidium* and *Giardia*, as well as viruses. *E. coli* can be transported through storm water after a heavy rainfall washing infected manure into the farming community’s wells and subsequently contaminating ground water and soil. Water contamination by *E. coli* is becoming common in rural areas of the United States, with up to 40% of tested wells found to be contaminated [4].

In 1999, hurricane Floyd caused serious flooding of swine waste in North Carolina, polluting surface water, and resulting in the contamination of the environment with fecal bacteria [4]. After the 2006 storm, new technologies such as constructed wetlands for treating waste from swine operations in the state came into effect [4]. The extent of water treatment in constructed wetlands depends upon the wetland design, microbial community, and types of plants involved, and a combination of these processes may help in the removal of nitrogen (N), phosphorus (P), solids, and chemical oxygen demand (COD) from treated swine wastewater, therefore, preventing the overloading of nutrients on agricultural land to which the effluent is applied [5]. The results from a subsequent study [6] showed that the bacterial colony forming units (CFU) and the average concentrations of total nitrogen, NH_4_^+^, total phosphorous (TP) and PO_3_^−^ from the influent to the effluent decreased. The NH_4_^+^ and the PO_4_^3−^ concentrations showed the most dramatic changes, with decreases of 39.97% and 16.92%, respectively. This followed a similar trend in a dairy wetland in southern California, USA [2]. The study in California provided good evidence of the effectiveness of wetland technology in water reclamation. In another study, Ibekwe et al. [3] showed that surface flow-constructed wetlands could serve as a model for waste management from concentrated animal feeding operations (CAFOs) and other confined-animal facilities, resulting in the general improvement of ground and surface water quality. The present study looks at *E. coli* serotype identification, population structure, and genetic diversity of *E. coli* in a surface flow-constructed wetland systems in association with continuous flow ponds. The addition of two continuous flow ponds is new and this has been suggested as a potential treatment option prior to land application. The main contaminants from swine waste may include nutrients, salts, microbes, and pharmaceutically active compounds and their removal involves complex physical, chemical, and biological processes. *E. coli* are widely used as indicators of fecal contamination of waterways in most urban and rural areas. *E. coli* have diverse genotypes and phenotypes, and some characteristics are shared among strains exposed to similar environments due to selection pressure [7]. Our main objectives were to describe the abundance and reduction of *E. coli* in different configurations of constructed wetlands and correlating these data to the quality of the final effluent. The overall goal of this wetland is to have the final effluent water that is suitable for on-site reuse and reduces the number of contaminants entering the environment.

## 2. Materials and Methods

### 2.1. Experimental Site and Sampling

The experimental site was a continuous flow-constructed wetland located at a swine research facility at North Carolina Agricultural and Technical State University farm in Greensboro, NC, USA (Figure S1). The wetland has six cells 40 m long by 11 m wide and was constructed in 1995 [5,8]. Each cell consisted of 11 m by 10 m marsh at both influent and effluent and 11 m by 20 m pond section separating the marshes and planted with *Typha latifolia* L. (broadleaf cattail) and *Scirpus americanus* (bulrush) in March 1996 [5]. The marsh and pond sections of wetlands have previously been described [6]. The number of pigs from January 2007 to January 2012 ranged between 65 and 115. Waste flow from the swine house was flushed with recycled water into a two-stage anaerobic lagoon, and the flow from the lagoon was pumped into a storage tank as described before [6]. The wastewater from the storage tank was discharged by gravity into the wetland cells, and the final effluent from the wetland was discharged into a holding pond for recycling into the swine house or application on land. To detect the spatial and temporal variation of *E. coli* populations in wetland effluent, water samples were collected from different points in April, August, and November 2010. All samples were maintained on ice until arrival in the laboratory and then stored at 4 °C for further analysis. Samples were analyzed for ammonia (NH^+^_4_–N), nitrate (NO_3_–N), total-P (TP) and available-P (PO_4_^3−^) using a flow injection analysis instrument (Lachat-QuikChem 8000, Loveland, CO, USA). The carbon (C) and nitrogen (N) concentrations were measured using a Perkin–Elmer 2400, CHNS/O series II Analyzer (Shelton, CT, USA).

### 2.2. Enumeration E. coli from MPN Method

All samples were analyzed using Colilert vessel (Westbrook, ME, USA) according to the manufacturers’ protocol, and *E. coli* population was expressed in Most Probable Number (MPN/100 mL). For isolation of *E. coli*, 100 μL liquid sample was removed from positive wells, then spread plated onto Chromagar ECC agar (CHROMagar Microbiology, Paris, France), and incubated at 37 °C for 24 h. All colonies were preserved at −80 °C for further characterization. Manure samples (10 g) were diluted with 90 mL of phosphate buffered saline (PBS) water (0.0425 g/L KH_2_PO_4_ and 0.4055 g/L MgCl_2_) and shaken for 15 min and individual colonies were processed as above according to method 9223 [9].

### 2.3. Isolation of Potential Pathogenic E. coli from Wetland

Harlequin cefixime-tellurite sorbitol MacConkey (CT-SMAC) agar with BCIG (5-bromo-4-chloro-3-indoxyl-β-d-glucuronide) (LAB M: IDG–Lancashire, UK) was used for the isolation of *E. coli* O157. All plates were incubated for 16 h at 37 °C. Translucent colonies (10) per sample were tested by multiplex PCR to determine the presence of *hyl*A, *stx*1, *stx*2, and *eae* genes [10]. Red/pink colonies with a purple center or green colonies) were enumerated as other *E. coli* or non O157 or presumptive pathogenic *E. coli.* All isolates were tested for heat labile toxin (LT), heat stable toxins a and b (STa and STb), Shiga toxins 1 and 2 (*stx*1 *and stx*2), cytotoxin necrotizing factors 1 and 2 (*cnf*1 and *cnf*2), intimin (*eae*), including O and H serotypes [11] Table 1).

### 2.4. Typing of E. coli Using REP-PCR

Genomic DNA fingerprinting of *E. coli* isolates was performed using a procedure described by [12,13,14]. Repetitive Extragenic Palindromic-PCR (REP-PCR) was used to assess the genetic diversity of *E. coli* isolates. Rep-PCR fingerprints were obtained by using primer REP 1R (5′-IIIICGICGICATCIGGC-3′) and REP 2I (5′-ICGICTTATCIGGCCTAC-3′) [15,16]. All images were visualized using quality one gel imaging system (Bio-Rad Lab., Hercules, CA, USA). All comparisons were done with the BioNumerics software, version 7.5 (Applied Maths, Austin, TX, USA). Fingerprints were clustered using the Jaccard coefficient evaluated by the unweighted-pair group method (UPGMA).

### 2.5. Analysis of E. coli Genotyping

*E. coli* isolates were analyzed temporally and spatially as previously described [13] using REP-PCR DNA fingerprinting. Briefly, the total number of unique *E. coli* genotypes was calculated, the distribution of the genotypes, and occurring frequencies in the wetland samples were determined using a Pearson similarity coefficient and UPGMA (unweighted pair-group method using arithmetic averages). Genotype accumulation curves, the unique genotypes, and their abundances (i.e., how many isolates share the same genotypes) were calculated and analyzed.

### 2.6. Statistical Analysis

Data analyses were done in duplicate using analysis of variance (ANOVA) with log_10_-transformed density of *E. coli* bacteria using SAS version 9.1 [17] to determine statistically significant differences, and Tukey’s studentized test range (HSD) was used for mean separation. Shannon diversity index (*H′*) was used to calculate genetic diversity as previously described [18]:

$$H\prime =-\sum_{i=1}^{S}p_{i}\ln p_{i}$$

where *S* is the number of unique genotypes and *p_i_* is the number of isolates sharing the same genotype, *i*, over the total number of isolates.

## 3. Results

### 3.1. Removal E. coli Isolates and Nutrients from Wetland Samples

**Figure 1:**
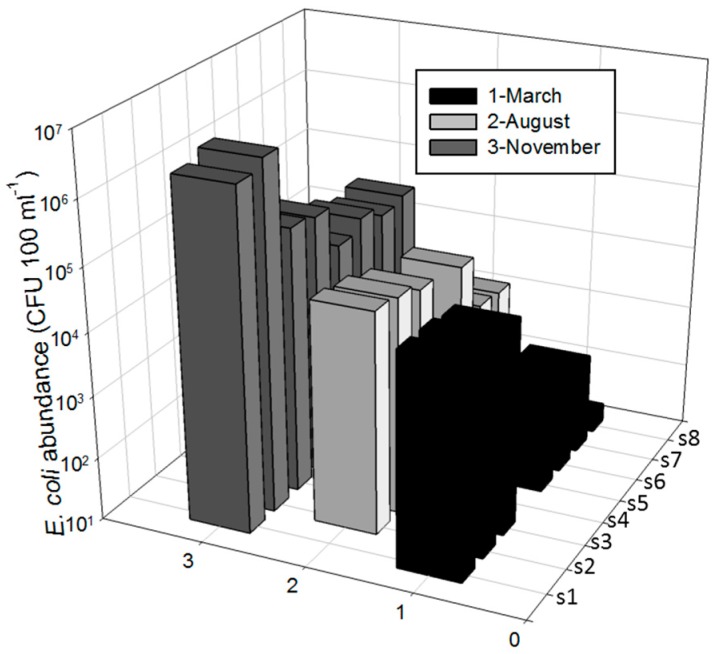
*E. coli* population in March, August, and November in wetlands. Symbols on the *X*-axis are effluent from swine house (S1), two-stage anaerobic lagoon system consisting of a primary lagoon 1 (S2) with overflow into a secondary lagoon 2 (S3) that flows to the storage tank (S4), continuous wetland cell influent (S5), continuous wetland cell effluent (S6), storage pond (S7), final effluent samples (S8) where it was recycled for flushing of the swine production facility and for land application). All samples were collected in duplicate.

To assess the spatial and temporal variations of *E. coli* population and genotypes isolates were obtained from manure effluent (S1) through wetland effluent (S8) during March, August, and November from continuous flow section of a marsh-pond-marsh constructed wetland (Figure S1). *E. coli* population was significantly (*p* = 0.036) higher in November than in March and August (Figure 1) in the pit finishing barn effluent (S1), lagoon 1 (S2, S3), and storage pond (S4). However, no differences were found in other cells. Spatially, and during November, there was a significant decline in *E. coli* populations from the manure effluent (S1) to the final effluent (S8). To determine spatial variation, 421 *E. coli* isolates obtained from waste water after processing in Colilert vessels (see Materials and Methods) were further processed for genotypic analysis using REP-PCR.

**Figure 2:**
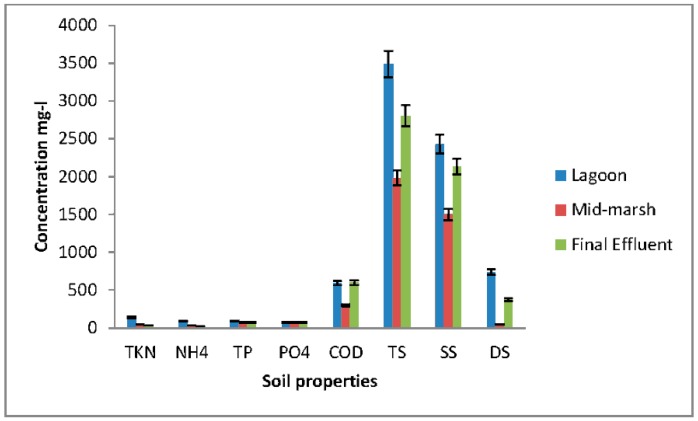
Nutrient removal in wetland samples collected in duplicate.

Total N, ammonium (NH_4_^+^), and total suspended solids (TS) significantly decreased (*p* < 0.05) in wetlands from the lagoon to the final effluent (Figure 2). About 70% of N and NH_4_^+^ were removed from the influent to the effluent. The removal rate was similar to that previously reported [5,19]. Removal efficiency of total and organic phosphorus and total suspended solids were significantly higher (*p* < 0.01) between lagoon and the final effluent.

### 3.2. Characterization of Potentially Pathogenic E. coli

A total of 421 *E. coli* isolates were used for all analysis. None of these strains was classified as Shiga toxin producing *E. coli* O157 (STEC). The rest were classified as enterotoxigenic *E. coli* (ETEC) due to the presence of heat labile or heat stable toxin-encoding genes. A total of eleven isolates (15%) carried *stx*2 gene, none carried *stx*1 and 20% of the isolates carried *eae* genes (The *eae*+ *stx*-isolates should be classified as enteropathogenic *E. coli*, EPEC). However, none of the isolates with *eae* genes carried *stx*2 genes. Therefore, none of these isolates were classified as STEC O157 or any other O groups based on the typing protocol. On the other hand, 72% of the isolates carried either of the heat stable toxins a and b (*sta*/*stb*) genes reflecting that these are ETEC strains. The high concentrations of ETEC suggest high prevalence of *E. coli* in swine manure, which may cause diarrhea. Therefore, treating swine waste water before release to the environment, is critical.

### 3.3. Diversity of E. coli Isolates in the Continuous Flow-Constructed Wetland with REP-PCR

**Figure 3:**
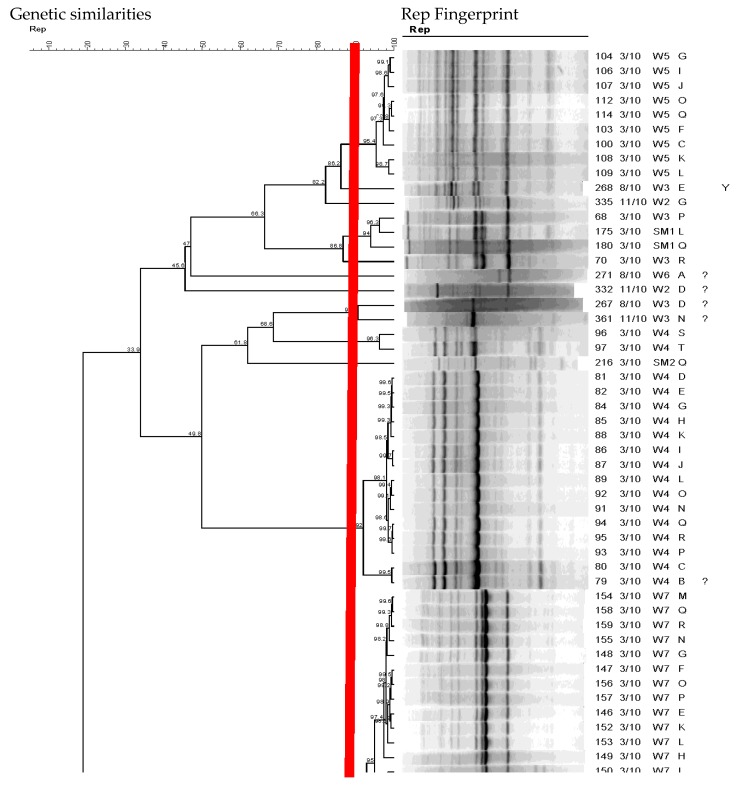
Dendrogram showing the genetic relatedness of *E. coli* from wetland based on their rep-PCR DNA fingerprints as an example. The red vertical line indicates the cutoff value for identifying unique genotypes.

Using a Pearson similarity coefficient and UPGMA strains with fingerprint patterns with similarity above 90% were considered clonal populations and were analyzed by REP-PCR DNA fingerprinting (Figure 3 with the dotted line representing the 90% cutoff point for unique genotypes).

**Figure 4:**
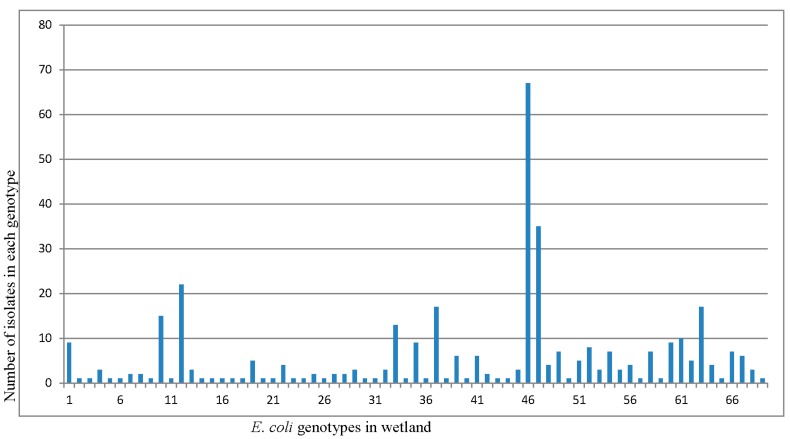
*E. coli* isolates (421) from swine wetland were analyzed by REP-PCR DNA fingerprint and then grouped into 69 unique genotypes based on cluster analysis. The distribution of genotypes among the sampling sites and their detection frequencies, i.e., number of isolates per genotype at different sites.

**Figure 5:**
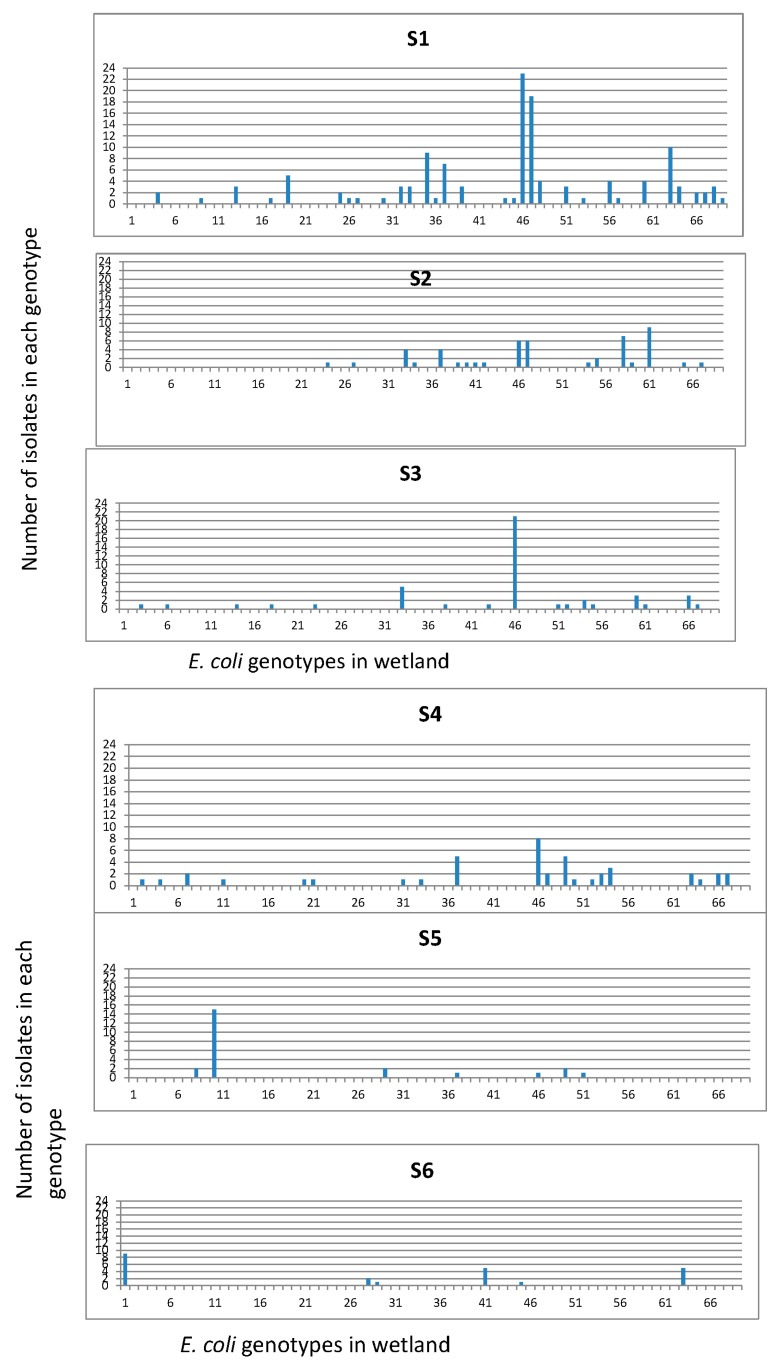

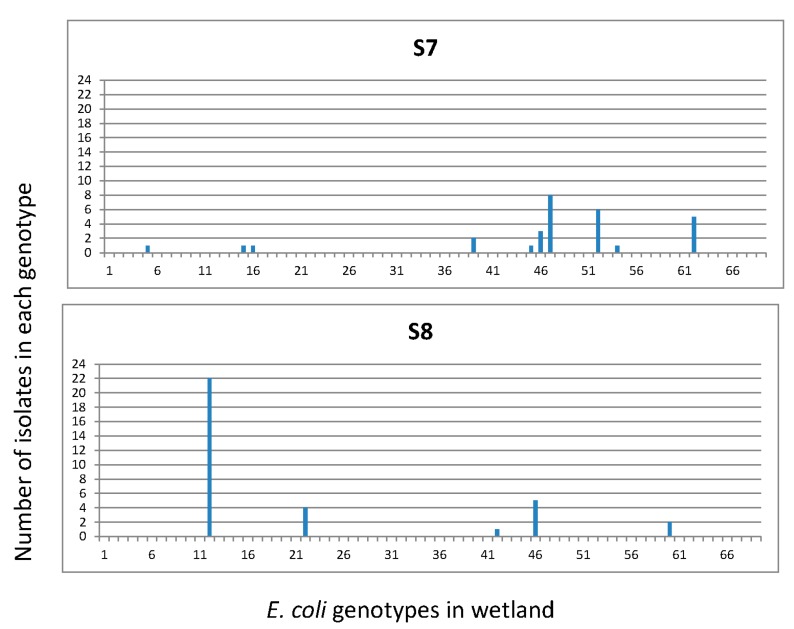
*E. coli* genotypes based on REP PCR distributions. Symbols on the *X*-axis are effluent from swine house (S1), two-stage anaerobic lagoon system consisting of a primary lagoon 1 (S2) with overflow into a secondary lagoon 2 (S3) that flows to the storage tank (S4), continuous wetland cell influent (S5), continuous wetland cell effluent (S6), storage pond (S7), final effluent samples (S8) where it was recycled for flushing of the swine production facility and for land application.

Isolates were grouped into 69 REP-PCR unique genotypes (Figure 4) with a Shannon diversity index (*H′*) of 3.231 (Table 2). The population comprised only three genotypes clustering more than 20 isolates, while 66 genotypes clustered between 2 and 19 isolates. The three dominant genotypes were consistently found at each sampling site and in every sampling month. The distribution of genotypes among the sampling sites and their detection frequencies, i.e., number of isolates per genotype at different sites were determined (Figure 5). The frequencies of obtaining a unique *E. coli* genotype, as indicated by ratios of genotypes vs. isolates (Table 3), were 0.23 for isolates from manure (S1), 0.31 for isolates from primary lagoon (S2), 0.30 for secondary lagoon isolates (S3), 0.42 for storage tank isolates (S4), 0.29 for continuous wetland influent isolates (S5), 0.20 for continuous wetland effluent isolates (S6), 0.27 for storage pond isolates (S7), and 0.15 for the final effluent isolates (S8).

**Figure 6:**
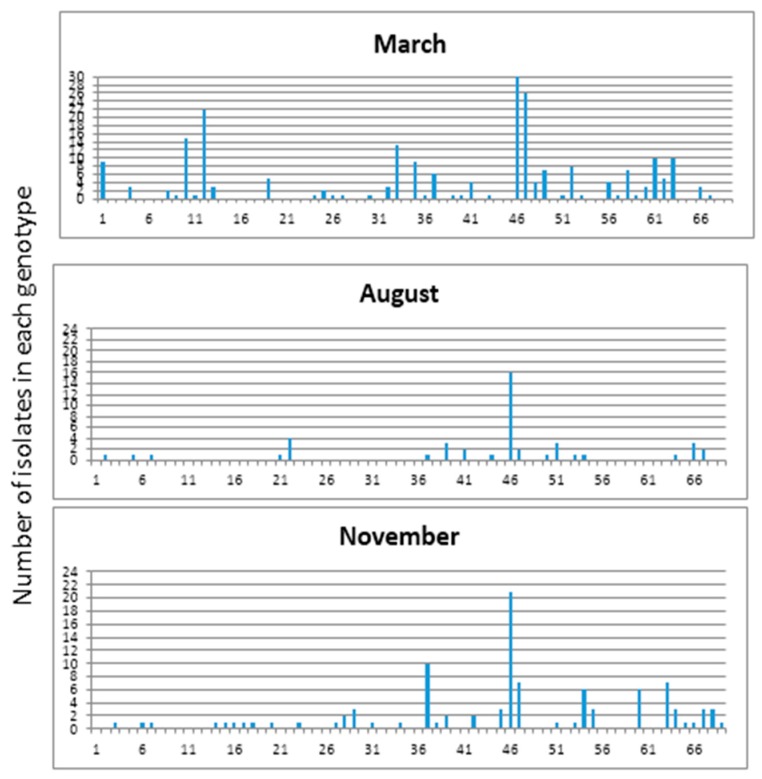
Temporal variations of *E. coli* isolates based on rep-PCR analysis in wetland for March, August, and November.

*E. coli* isolates collected during March, August, and November from manure and wetland effluent showed temporal variation based on REP-PCR (Figure 6 Unique *E. coli* genotype frequencies obtained in the study were 0.16, 0.28 and 0.30 for isolates collected in March, August, and November, respectively. In March 247 isolates with 40 unique genotypes were obtained with genetic diversity (*H′*) of 3.173. Whereas in November only 113 isolates with 34 unique genotypes, and *H′* of 3.019 were detected. However, in August only 61 isolates with 18 unique genotypes and a Shannon diversity index (*H′*) of 2.335 could be detected. (Table 3). It should be noted that each genotype in March contained higher numbers of isolates than what were observed in August and November indicating fewer clonal populations in March.

## 4. Discussion

The analysis of virulence factors did not show the presence of the typical ETEC virulent genes; *elt*, *est*B, and *fae*G. However, many *E. coli* strains isolated carried heat-stable enterotoxin a and b (STa and STb) genes which are very common in ETEC (Table 1). Based on the analysis and confirmation tests, none of our isolates was classified as *E. coli* O157. Other studies have shown high prevalence of STa and STb genes from *E. coli* samples isolated from swine [20] and greater reduction of potentially pathogenic *E. coli* from waste water treatment [21]. Removal efficiencies of microbes from constructed wetlands have been shown to be based on the type of wetland plants employed [2,3].

Microorganisms in densities above certain levels in water can cause adverse effects, including death in humans and wildlife as a result of exposure. Adverse health effects in humans can be grouped into gastrointestinal, respiratory, eye, ear, nose, skin rashes, etc. *E. coli* counts were significantly (*p* = 0.01%) different among the three month flows. Others have reported the typical enteric bacteria removal between 1 and 3 log_10_ from constructed wetlands [22,23,24,25]. Data from this study agree with the above studies from the wetland described [5,6,19,22]. Other studies using the same wetland associated the decreased bacterial counts with spatial nutrient content differences in the wetland where the concentrations of TN, NH^+^_4_, TP and PO^3−^_4_ decreased from influent to effluent of the wetland [19,22]. We previously showed a 98% decrease in *E. coli* with subsurface constructed wetland, and this reduction was significantly correlated with TN, NH^+^_4_, TP and PO^3−^_4_ decreased from influent to effluent in the wetland [2]. Although the number of swine was relatively low in this wetland, it provided us with the opportunity to study *E. coli* population dynamics from wetland cell to wetland cell and to document which section of the wetlands are more efficient in waste removal. The overall results could be partly explained that N and P are essential nutrients for bacterial growth and the decrease in these nutrients probably had a role in *E. coli* population decrease. Another reason may be due to die-off following removal from swine gut and exposure to environment, together with predation by filter feeders, and entrapment within plant/soil matrix of CW. The reduction in *E. coli* population also is in agreement with microbial community study from the same wetland [8] that showed that community structures from wetland samples associated with the swine house (S1) and lagoons (S2, S3) were significantly different (*p* = 0.0001) from storage tanks (S4), wetland cells effluent (S5), storage pond (S7), and the final effluent (S8). The most significant reduction started occurring in mid mash wetland cells (S5) where there were likely interactions of microbial activities with wetland plants. In this study, the concentration of *E. coli* in the final effluent (S8) was significantly lower especially during. Therefore, the trend seems to follow a similar pattern in bacterial removal whether at the single isolate level or at the microbial community level.

In this study, we found that *E. coli* populations between the wetland influent and effluent were significantly reduced. The reduction in bacterial counts by constructed wetland means much lower bacteria loadings to the environment [26] and may result in significant reduction of bacterial counts [27]. Constructed wetlands are used to reduce bacterial concentrations from possible transfer from storage ponds through irrigation water to agricultural fields or to drinking water sources. The great threat of bacterial contamination to drinking water may be the high concentrations of such bacteria in the source water that may result in the transfer of genetic elements from nonpathogenic to pathogenic strains. Pathogens with increased resistance to different antimicrobials may easily be transported from animal manure to rivers during through surface runoff or even to groundwater through leaching [28]. In developing countries the water from such river may be a source for domestic water supply. It has also been reported that changes in *E. coli* composition in surface water could be a consequence of seasonal changes with summer populations derived from numerous sources than winter populations [14,29]. *E. coli* population can also undergo changes during the lifetime of the animals due to changes in diet which may also vary with seasons. In this study, higher diversity was observed in summer and autumn months (August and November) than during the winter month (March), as these changes could be related to changes in diet during this period.

In this study, March samples showed higher numbers of isolates in each genotype than in August and November (Figure 5). This may be due to higher numbers of clonal populations during the winter months. Changes in *E. coli* diversity associated with seasons had been well documented in water communities [13,30,31], but little has been done in constructed wetlands. In this study, many environmental *E. coli* isolates were systematically obtained from the wetlands to study the spatial and temporal variations and overall genotypic diversities of *E. coli* from different sections of the wetlands. Temporally, the genotypic compositions of *E. coli* for the three sampling times based on REP-PCR were very different from our previous study based on BOX-AIR PCR [22]. Individual water samples obtained from each sampling time contained several dominant genotypes with high abundances and many less dominant genotypes with lower abundances.

Interestingly, higher numbers of isolates were observed in each genotype from mid marsh to the final effluent. This suggests fewer dominant population of *E. coli* from the mid marsh to the final effluent. The same dominant *E. coli* genotypes were also observed at different locations in rivers affected by agricultural [14] and urban [32] (McLellan 2004) land uses. The main reason for some of the dominant genotype may be the presence of a dominant point fecal source from the constructed wetland originating from the swine fecal materials.

## 5. Conclusions

In summary, the significant reduction of *E. coli* between influent and effluent water in this study may be due to the reduction of different nutrients in different sections of the wetland or die off in the environment. The reduction of *E. coli* is a significant example of the potential reduction of pathogens that may enter surface water via animal waste. The removal of the main pollutants from the swine waste would have a beneficial impact on the surface and groundwater in many rural areas of North Carolina with swine production, and in turn, would benefit the quality of water leading into rivers. The findings from this study and similar studies will aid with protecting our surface and ground waters and may reduce the outbreak of severe infections, because most of these diseases are caused by typical water related pathogens. Therefore, the use of continuous flow-constructed wetland for water quality improvements must be encouraged at all levels. This is critical especially in developing countries where resources are limited, and pollution from fecal material is high.

## Figures and Tables

**Table 1:** Virulence gene and resistant genotypes of potential pathogenic *E. coli* from swine constructed wetland.

Month	Sample Name	O Type	H Type	LT	Sta	STb	STX1	STX2	EAE	CNF1	CNF2
March	S1C	-	43	-	-	-	-	-	-	-	-
March	S1F	-	43	-	-	-	-	-	-	-	-
March	S1G	-	43	-	-	-	-	-	-	-	-
March	S1U	-	19	-	+	+	-	-	-	-	-
March	S1W	-	4	-	+	+	-	-	-	-	-
March	S2C	-	11	-	-	-	-	-	+	-	-
March	S2D	-	11	-	-	-	-	-	+	-	-
March	S2E	-	11	-	-	-	-	-	+	-	-
March	S2K	-	43	-	-	-	-	-	-	-	-
March	S2P	88	38	-	-	-	-	-	-	-	-
March	S2Y	-	4	-	+	+	-	-	-	-	-
March	S2Z	-	4	-	+	+	-	-	-	-	-
March	S2AA	-	4	-	+	+	-	-	-	-	-
March	S2AB	-	4	-	+	+	-	-	-	-	-
March	S2AC	-	4	-	+	+	-	-	-	-	-
March	S3W	-	4	-	+	+	-	-	-	-	-
March	S3X	-	9	-	+	-	-	-	-	-	-
March	S3Y	-	4	-	+	+	-	-	-	-	-
March	SM1A	-	36	-	+	+	-	+	-	-	-
March	SM1C	-	36	-	+	+	-	+	-	-	-
March	SM1T	-	36	-	+	+	-	+	-	-	-
March	SM1U	-	36	-	+	+	-	+	-	-	-
March	SM1V	-	36	-	+	+	-	+	-	-	-
March	SM2N	98	5	-	-	+	-	-	-	-	-
March	SM2U	-	36	-	+	+	-	+	-	-	-
March	SM2V	-	36	-	+	+	-	+	-	-	-
March	SM2W	-	11	-	-	-	-	-	+	-	-
March	SM2X	-	36	-	+	+	-	+	-	-	-
March	SM2Y	-	11	-	-	-	-	-	+	-	-
March	SM2Z	-	19	-	+	+	-	-	-	-	-
March	PW1A	-	19	-	+	+	-	-	-	-	-
March	PW1B	-	19	-	+	+	-	-	-	-	-
March	PW1C	-	19	-	+	+	-	-	-	-	-
March	PW1D	-	19	-	+	+	-	-	-	-	-
March	PW2A	-	19	-	+	+	-	-	-	-	-
March	PW2B	-	19	-	+	+	-	-	-	-	-
March	PW2C	-	19	-	+	+	-	-	-	-	-
March	PW2D	-	19	-	+	+	-	-	-	-	-
March	PW2E	-	19	-	+	+	-	-	-	-	-
March	PW3A	-	19	-	+	+	-	-	-	-	-
March	PSM2E	-	19	-	+	+	-	-	-	-	-
March	PSM2F	-	19	-	+	+	-	-	-	-	-
March	PSM2G	-	19	-	+	+	-	-	-	-	-
March	PSM2H	-	19	-	+	+	-	-	-	-	-
March	PSM2I	-	19	-	+	+	-	-	-	-	-
March	PSM2J	-	19	-	+	+	-	-	-	-	-
March	PSM2K	-	19	-	+	+	-	-	-	-	-
March	PSM2L	-	19	-	+	+	-	-	-	-	-
March	PSM2M	-	19	-	+	+	-	-	-	-	-
March	PSM2N	-	19	-	+	+	-	-	-	-	-
March	PSM2O	-	19	-	+	+	-	-	-	-	-
March	PSM2P	-	19	-	+	+	-	-	-	-	-
August	S1C	-	11	-	-	-	-	-	+	-	-
August	S1H	-	11	-	-	-	-	-	+	-	-
August	S1J	-	11	-	-	-	-	-	+	-	-
August	S1K	-	11	-	-	-	-	-	+	-	-
August	S2B	-	32	-	-	-	-	-	-	-	-
August	S3E	178	+	-	-	-	-	-	-	-	-
November	S1L	-	11	-	-	-	-	-	+	-	-
November	S2K	-	11	-	-	-	-	-	+	-	-
November	S2L	-	11	-	-	-	-	-	+	-	-
November	S3A	-	+	-	-	-	-	-	-	-	-
November	S3B	2	+	-	-	-	-	-	-	-	-
November	S3C	-	4	-	-	-	-	-	-	-	-
November	S3G	-	11	-	-	-	-	-	+	-	-
November	S3H	-	11	-	-	-	-	-	+	-	-
November	S3J	-	11	-	-	-	-	-	+	-	-
November	S3O	-	4	+	+	-	-	-	-	-	-
November	S6K	-	11	-	-	-	-	-	+	-	-
November	SM1G	-	30	-	+	-	-	+	-	-	-
November	SM1H	-	30	-	+	-	-	+	-	-	-
November	SM1L	-	30	-	+	-	-	+	-	-	-

- means not detected and + means detected.

**Table 2:** Temporal variations of Shannon diversity indices (*H*′) of *E. coli* isolate based on BOX PCR.

Season	No. of Isolates	No. of Unique Genotypes	*H′* Index	Frequency Ratio
March	247	40	3.173	0.16
August	61	18	2.335	0.28
November	113	34	3.019	0.30
Total	421	92	3.231	0.25

**Table 3:** Shannon diversity indices (*H*′) of *E. coli* isolates based on BOX PCR from different locations within the wetland.

Sampling Locations	No. of Isolates	No. of Unique Genotypes	*H′* Index	Frequency Ratio
swine house effluent (S1)	135	31	2.976	0.23
primary lagoon 1 (S2)	57	18	2.569	0.31
secondary lagoon 2 (S3)	57	17	2.177	0.30
storage tank (S4)	48	20	2.779	0.42
continuous wetland influent (S5)	24	7	1.310	0.29
continuous wetland effluent (S6)	30	6	1.705	0.20
storage pond (S7)	36	10	2.090	0.27
land application (S8)	34	5	1.086	0.15

## Supplementary Materials

The following are available online at http://www.mdpi.com/1660-4601/15/7/1369/s1.
