# Supplementary material for: Continuous Flow-Constructed Wetlands for the Treatment of Swine Waste Water
Source: Int J Environ Res Public Health. 2018 Jun 29;15(7):1369. doi: 10.3390/ijerph15071369 (PMC6068887; doi:10.3390/ijerph15071369)
Supplement: Supplementary file 1 [file ijerph-15-01369-s001.pdf]

Material:

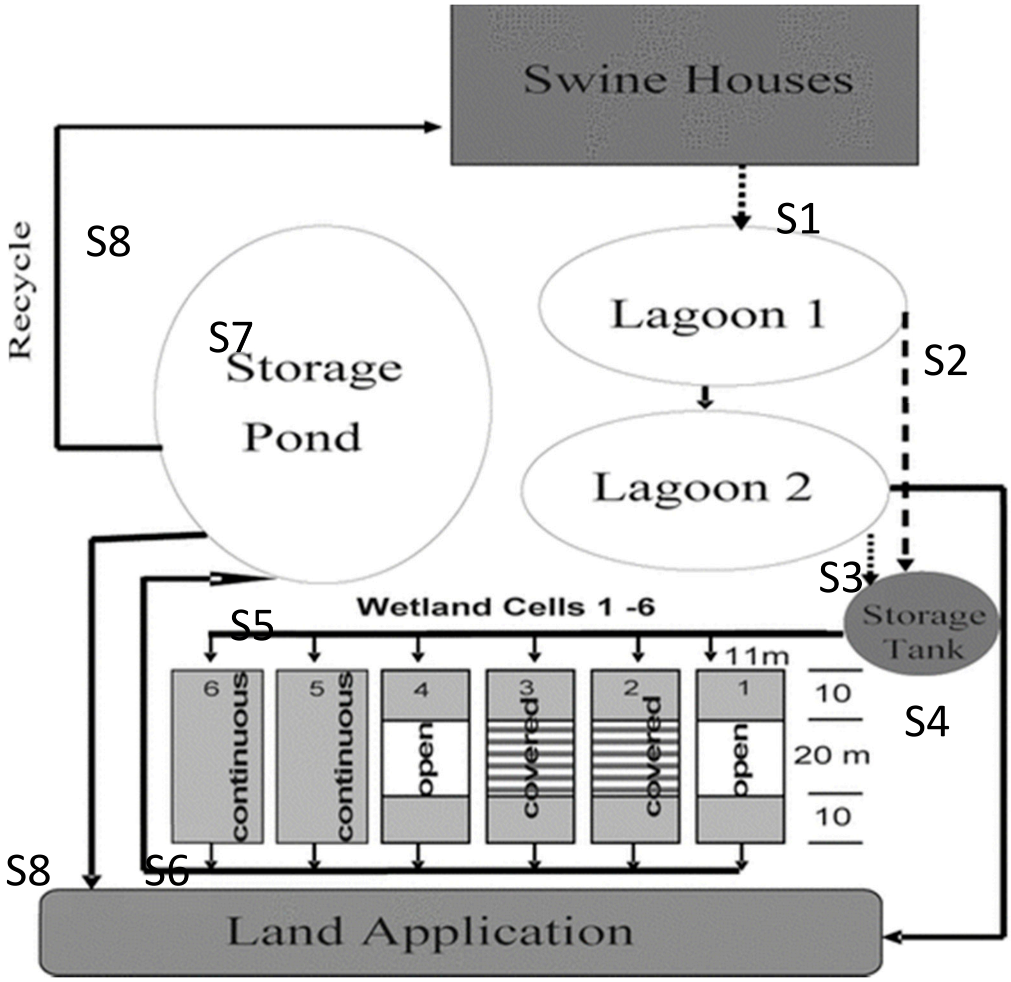

Figure S1. Constructed wetland located at a swine research facility at North Carolina Agricultural and Technical State University farm in Greensboro, NC, USA. S1 indicates effluent from the from the swine house into a two-stage anaerobic lagoon system consisting of a primary lagoon 1 (S2) with overflow into a secondary lagoon 2 (S3) that flows to the storage tank (S4). Wastewater flows from the 8000 L storage tank by gravity to each continuous wetland cell influent for sampling point S5. The final effluent samples (S6) from the continuous marsh cells are discharged into a storage pond (S7) and recycled for flushing of the swine production facility and for land application (S8).
